# Utility and feasibility of integrating pulse oximetry into the routine assessment of young infants at primary care clinics in Karachi, Pakistan: a cross-sectional study

**DOI:** 10.1186/s12887-015-0463-z

**Published:** 2015-09-30

**Authors:** Connor A. Emdin, Fatima Mir, Shazia Sultana, AM Kazi, Anita K M Zaidi, Michelle C. Dimitris, Daniel E. Roth

**Affiliations:** Department of Pediatrics and Centre for Global Child Health, The Hospital for Sick Children, Toronto, ON Canada; Department of Pediatrics and Child Health, The Aga Khan University, Karachi, Pakistan; Department of Pediatrics, University of Toronto, Toronto, ON Canada; The Hospital for Sick Children, 686 Bay Street, Toronto, ON M5G 0A4 Canada

## Abstract

**Background:**

Hypoxemia may occur in young infants with severe acute illnesses or congenital cardiac anomalies, but is not reliably detected on physical exam. Pulse oximetry (PO) can be used to detect hypoxemia, but its application in low-income countries has been limited, and its feasibility in the routine assessment of young infants (aged 0–59 days) has not been previously studied. The aim of this study was to characterize the operational feasibility and parent/guardian acceptability of incorporating PO into the routine clinical assessment of young infants in a primary care setting in a low-income country.

**Methods:**

This was a cross-sectional study of 862 visits by 529 infants at two primary care clinics in Karachi, Pakistan (March to June, 2013). After clinical assessment, oxygen saturation (Sp02) was measured by a handheld PO device (Rad-5v, Masimo Corporation) according to a standardized protocol. Performance time (PT) was the time between sensor placement and attainment of an acceptable PO reading (i.e., stable SpO_2_ + 1 % for at least 10 s, heart rate displayed, and adequate signal indicators). PT included the time for one repeat attempt at a different anatomical site if the first attempt did not yield an acceptable reading within 1 min. Parent/guardian acceptability of PO was based on a questionnaire and unprompted comments about the procedure. All infants underwent physician assessment.

**Results:**

Acceptable PO readings were obtained in ≤1 and ≤5 min at 94.4 % and 99.8 % of visits, respectively (n = 862). Median PT was 42 s (interquartile range 37; 50). Parents/guardians overwhelmingly accepted PO (99.6 % overall satisfaction, n = 528 first visits). Of 10 infants with at least one visit with Sp02 <92 % on a first PO attempt, 3 did not have a significant acute illness on physician assessment. There were no PO-related adverse events.

**Discussion:**

Using a commercially available handheld pulse oximeter, acceptable Sp02 measurements were obtained in nearly all infants in under 1 minute. The procedure was readily integrated into existing assessment pathways and parents/guardians had positive views of the technology.

**Conclusions:**

When incorporated into routine clinical assessment of young infants at primary care clinics in a low-income country, PO was feasible and acceptable to parents/guardians. Future research is needed to determine if the introduction of routine PO screening of young infants will improve outcomes in low-resource settings.

## Background

Timely management of severe neonatal infections (e.g., sepsis, pneumonia) is a crucial component of public health strategies to reduce infant mortality in low-income regions [1]. Although community health workers (CHWs) can be trained to recognize signs of illness in young infants (aged 0 to 59 days), appropriate triage and referral of the most critically ill infants relies on the recognition of signs of early or impending cardiorespiratory failure, including hypoxemia [2, 3]. Existing clinical algorithms based on signs and symptoms, such as the World Health Organization (WHO)/UNICEF Integrated Management of Childhood Illness (IMCI) approach, are insensitive for detecting hypoxemia in infants and young children [4].

Pulse oximetry (PO) is an non-invasive method of measuring peripheral oxygen saturation (SpO2) based on the differential absorption of red versus infrared light by oxygenated hemoglobin in a narrow tissue segment (e.g. infant’s hand or foot) [5]. The use of PO to evaluate cardiorespiratory function is nearly universal in health care settings in high-income countries, and has been accepted as a ‘fifth vital sign’ in pediatrics [6, 7]. Although there are few published studies of the added clinical value of routine PO in pediatric ambulatory or emergency care settings, studies in the US have provided evidence that routine PO may enable more effective triaging of children presenting with lower respiratory tract infections [8, 9]. Pulse oximetry has also recently been shown to be effective as a screening tool for early detection of critical congenital heart disease [10].

In primary care clinics where acute illnesses are triaged and managed, PO may be particularly useful in young infants (<2 months of age), among whom clinical features of serious illness are often subtle and non-specific. However, PO has infrequently been implemented in primary care clinics in low-income countries due to a range of both real and perceived barriers, including the availability and field robustness of low-cost PO devices, the time required to perform PO, and the challenges of accurate interpretation of PO readings by minimally-trained personnel (e.g., identification of motion artifacts or rejection of a spurious result due to a poor signal) [7]. Although there is emerging evidence that use of PO coupled with supplemental oxygen availability can improve hospital care delivery and outcomes, there is limited published experience related to the use of PO in the routine assessment of young infants (<2 months of age) in primary care clinics in low-income countries [11]. In the present study, we assessed the implementation of PO as a triage tool for young infants assessed at primary care clinics in Karachi, Pakistan. The aim was to determine if integration of PO into the routine assessment of young infants by CHWs is a practice that can be feasibly operationalized, is acceptable to parents/guardians, and provides clinical value.

## Methods

### Study setting and design

We conducted a cross-sectional facility-based study between March 5^th^ and June 1st, 2013 at two primary care clinics in low-income communities of Karachi, Pakistan. The Bilal Colony (Site 1) primary care clinic serves a population of 70 000, with approximately 40 young infant visits per week. The Bhains Colony (Site 2) primary care clinic serves a population of approximately 40 000, with 30 young infant visits per week. Both clinics provide well-child care (vaccination, growth monitoring, nutrition and hygiene education) and outpatient care for common childhood illnesses. A team of community health workers add outreach capacity through regular visits to households to detect early symptoms of illness such as newborn sepsis, and refer ill infants to these clinics for physician assessment. Seriously ill infants and children are provided transport to hospital or in case of refusal, centre-based parenteral antibiotic therapy (if indicated). Both sites are approximately located at sea level.

Study procedures were performed by a pair of study personnel – a study worker and a research assistant. Each clinic site was served by a core team of two study workers and one research assistant. Less than 2 % of the visits involved other trained personnel substituting for a core team member. “Study workers” refer to the four personnel (two at each clinic) who directly assessed infants according to the IMCI algorithm, performed pulse oximetry and conducted the initial infant parents/guardian interview. These individuals had secondary school education and prior experience in health research projects involving infants and children, but they did not have professional research or health care credentials. We considered their level of training/experience to be similar to that of a CHW. ‘Research assistants’ (one at each clinic) coordinated study activities, observed study workers to record data related to timing of pulse oximetry, and conducted an exit interview with parents/guardians. Research assistants had post-secondary education and had long-standing professional involvement in research as employees of Aga Khan University. All personnel were trained in formal sessions as well as a pilot implementation phase. None of the team members had used a pulse oximeter prior to this training/pilot period.

All visits by infants 0 to 59 days of age were eligible for inclusion, unless the infant was attending the clinic solely for a scheduled injectable antibiotic administration based on a previous diagnosis of suspected bacterial infection, or was too sick to undergo study procedures (because of the risk of delaying medical care). Infants were initially assessed by a Lady Health Worker (LHW, a government trained health worker) according to routine clinic intake procedures, including measurement of weight and axillary temperature. Eligible infants were then referred to the PO study team who obtained informed consent from the parent or guardian (that is, the infant’s caregiver in place of a parent) prior to proceeding with study procedures. Critically ill infants were referred directly to a clinic physician. Infants with previous study visits were eligible for an unlimited number of ‘revisits’ (i.e., second and subsequent visits).

The protocol was conducted in compliance with the Declaration of Helsinki and the protocol was approved research ethics committees at the Aga Khan University (Protocol No. 2006-Ped-ERC-11) and the Hospital for Sick Children (Protocol No. 1000028096). Written informed consent was obtained from all parents/guardians.

### Procedures

A structured parent/guardian interview was conducted by the study worker to elicit sociodemographic information, reason for clinic visit, and the parent/guardian perception of the infant’s illness severity. Enrolled infants underwent clinical assessment by the study worker according to the IMCI protocol, including enquiry about a history of convulsions or poor feeding, measurement of axillary temperature (if not already done by LHW) to determine presence of fever (>37.5 °C), counting of respiratory rate (RR) over 1 min (fast breathing defined as RR ≥ 60), observation for the presence of lower chest wall in-drawing, and determination of the level of consciousness/movement [3, 12].

Following the IMCI assessment, conducted by the study worker, SpO2 was measured using a commercially-available handheld PO device widely used in pediatric practice (Rad-5v, Masimo Corporation, Irvine, California), applying the low noise cabled sensor (LNCS) YI sensor (Masimo Corporation, Irvine, CA) to a left foot (first attempt) or right palm (second attempt, if needed) and secured in place using a reusable foam wrap, according to the manufacturer’s recommendation. First, the sensor (connected to the oximeter via an extension cable) was placed on the infant and subsequently, the oximeter was powered-on, an approach similar to that which has been previously referred to as “sensor to infant first” [13]. The Sp02 reading was considered “acceptable” if the measured SpO_2_ was stable (±1 %) for at least 10 s, the heart rate was displayed during the period of SpO_2_ measurement and the device’s functional indicators (‘blip bars’) suggested that the signal strength was adequate (i.e., green signal). If 1 min passed without obtaining an acceptable reading, the sensor was moved to the alternate site and the PO measurement was re-attempted. If five minutes passed without obtaining an acceptable reading, the measurement was considered attempted but unsuccessful. If an acceptable Sp02 was <90 %, the measurement was repeated to confirm the low value. Infants with Sp02 <90 % were offered referral to a tertiary health facility, regardless of the presence of other clinical signs. While the study worker performed the PO procedure, a research assistant observed and recorded the procedure time, Sp02, heart rate, and signal strength indicators displayed at the time the reading was deemed to be acceptable by the study worker. The study worker also recorded any spontaneous verbal comments by parents/guardians regarding the procedure (e.g., concerns about infant discomfort).

Infants were subsequently referred to a clinic physician, who completed a history and physical examination, and determined whether the infant required clinic-based treatment or immediate referral to hospital. To the extent that it was possible, the physician was not informed of the Sp02 prior to the determination of hospital referral. For 8 of 862 readings (0.9 %), blinding was not achieved, such that the physician knew of the SpO_2_ reading before making a decision regarding the need for hospital referral. Five of the 8 unmasked readings had SpO2 <90 %; in these cases, the physician was informed of the PO results to expedite clinical management. After completion of clinical and PO procedures, parents/guardians were asked to participate in a brief structured interview regarding perceptions and acceptance of PO.

Following the initial standardized PO procedure with the Rad-5v, PO was repeated using one of three other handheld PO devices: Lifebox (Acare Technology Co. Ltd., Taipei City, Taiwan), Tuffsat (General Electric Corporation, Fairfield, CO), or Nellcor OxiMax N-65 (Covidien Corporation, Mansfield, MA). The original intention was to establish feasibility on a variety of hand-held oximeters; however, we made a post-hoc decision to exclude data for these additional devices from the present analysis, for several reasons: these devices were each used less frequently than the Rad-5v such that personnel did not have comparable time to develop comfort and competence with their use; the secondary devices were not consistently used in both clinics and therefore were used by different personnel and among different patient populations (Table 1); measurements with the Rad-5v device always preceded the use of the secondary devices, such that infants may have been relatively more unsettled and personnel may have felt rushed during this second set of PO measurements.

**Table 1:** Characteristics of young infants undergoing routine pulse oximetry at initial study visits and revisits at primary care clinics in Karachi, Pakistan, stratified by study site

Characteristic	Total		Initial visits			Revisits		
	Initial visits	Revisits	Site 1	Site 2	*p*	Site 1	Site 2	*p*
Number of visits (*n*)	529	333	299	230		182	151	
Sex								
Male	267 (50.5 %)	186 (55.9 %)	153 (51.2 %)	114 (49.6 %)	*p=0.71*	103 (56.6 %)	83 (55.0 %)	*p=0.77*
Female	262 (49.5 %)	147 (44.1 %)	146 (48.8 %)	116 (50.4 %)		79 (43.4 %)	68 (45.0 %)	
Age								
0-6 days	177 (33.5 %)	32 (9.6 %)	123 (41.1 %)	54 (23.5 %)	*p<0.001*	18 (9.9 %)	14 (9.3 %)	*p=0.12*
7-29 days	176 (33.3 %)	160 (48.0 %)	95 (31.8 %)	81 (35.2 %)		96 (52.8 %)	64 (42.3 %)	
30-59 days	176 (33.3 %)	141 (42.3 %)	81 (27.1 %)	95 (41.3 %)		68 (37.4 %)	73 (48.3 %)	
Visit reason^a^								
Well baby visit^b^	97 (18.3 %)	26 (7.8 %)	77 (25.8 %)	20 (8.7 %)	*p<0.001*	22 (12.1 %)	4 (2.7 %)	*p<0.001*
Referred for new illness	424 (80.2 %)	209 (62.8 %)	222 (74.3 %)	202 (87.8 %)		154 (84.6 %)	55 (36.4 %)	
Follow-up visit for illness	8 (1.5 %)	72 (21.6 %)	0 (0 %)	8 (3.5 %)		6 (3.3 %)	91 (60.3 %)	
Parent/guardian’s perception of infant’s illness severity^c^								
Healthy	101 (19.1 %)	26 (7.8 %)	79 (26.4 %)	22 (9.6 %)	*p<0.001*	22 (12.1 %)	4 (2.7 %)	*p<0.001*
Minor illness	406 (76.8 %)	288 (86.5 %)	203 (67.9 %)	203 (88.3 %)		145 (79.7 %)	143 (94.7 %)	
Very sick	6 (1.1 %)	8 (2.4 %)	5 (1.7 %)	1 (0.4 %)		7 (3.9 %)	1 (0.7 %)	
Life-threatening	2 (0.4 %)	0 (0 %)	2 (0.7 %)	0 (0 %)		0 (0 %)	0 (0 %)	
Physician recommended referral to hospital?								
Yes	62 (11.7 %)	48 (14.4 %)	22 (7.4 %)	40 (17.4 %)	*p=0.001*	19 (10.4 %)	29 (19.2 %)	*p=0.03*
No	467 (88.3 %)	285 (85.6 %)	277 (92.6 %)	190 (82.6 %)		163 (89.6 %)	122 (80.8 %)	

^a^ *n* = 150 because one infant’s reason for a Site 2 revisit was missing

^b^Well baby visit refers to a visit for well-child care (vaccination, growth monitoring, nutrition and hygiene education)

^c^Parent/guardian perceptions of infant illness severity were missing at 25 visits (2.9 %)

### Outcome measures

The primary feasibility metric was the “performance time” (PT), in seconds, defined as the total time required to obtain an acceptable reading from the initiation of the procedure (when the study worker first picked up the sensor to place it on the infant) until an acceptable reading was obtained (study worker recorded a SpO_2_ value on the data form). The ‘lag time’ (LT), in seconds, was defined as the period from pressing the power button on the device until an acceptable reading was obtained. Whereas PT incorporated both personnel and device performance factors, the LT (a component of PT) primarily reflected device factors. Both PT and LT included the 10 s required to confirm that the displayed Sp02 was stable (criterion for acceptable reading). Parent/guardian perceptions of PO were measured by yes/no responses to a series of prompted items regarding overall satisfaction, openness to PO in the future, perceived usefulness, and concerns about infant discomfort. Unprompted parent/guardian concerns were also included in acceptability analyses.

To summarize acceptable Sp02 values, we considered that if the measurement was repeated because the initial attempt yielded an acceptable Sp02 <90 %, and the second attempt also yielded an acceptable reading, the higher of the two acceptable Sp02 values was included in descriptive analyses. However, the time to the first acceptable reading was used in analyses of the feasibility outcomes (PT and LT). In the original design of this study, we had aimed to explore the clinical utility of PO through analyses of the association of hypoxemia (Sp02 <90 %, per the WHO threshold [14]) with infant clinical outcomes; however, the prevalence of hypoxemia was much lower than anticipated, and such analyses were not feasible within the current dataset. Instead, as a post-hoc descriptive analysis, we summarized the clinical information for infants with any acceptable Sp02 <92 % (a threshold for hypoxemia often used in clinical practice [15]) at any visit, including the range of Sp02 at all the infants’ visits, IMCI assessment by the study worker and physician diagnosis at the visit at which hypoxemia was first detected, and vital status at 2 months of age (if available).

### Statistical analysis

Descriptive statistics included median (interquartile range, IQR) for non-normally-distributed variables and proportions for dichotomous/categorical variables. We considered feasibility in terms of the median PT and LT, as well as the proportion of acceptable readings obtained within 1 min (primary target) and 5 min. We investigated whether the feasibility outcomes (PT and percent acceptable readings within 1 min) were significantly associated with any of the following factors: clinic site, day of visit relative to the start of the study, age, sex, weight, reason for visit and presence/absence of at least one IMCI criteria. Differences in PT across strata (e.g., between age groups,) were assessed using non-parametric testing (Wilcoxon rank-sum tests). Associations between PT and continuous covariates (e.g., age) were also analyzed using Spearmen’s rank correlation coefficient. Differences in proportions (i.e. % of readings obtained within 1 min, baseline participant characteristics and parent/guardian acceptability metrics) were analyzed by Chi-squared tests. Because clinic site was a strong predictor of PT, analyses for other factors were performed after stratification by site. Among infants with >1 visit during the study period, there was no correlation between PT at initial and first revisits (Spearmen’s Rho = 0.0518, p = 0.4938, n = 177). Therefore, visits by the same infant were treated independently in analyses of feasibility metrics.

## Results

Of 1166 visits screened for eligibility during the study period, 1084 (93 %) were eligible for inclusion and 862 (74 %) were enrolled. Of 222 ineligible visits, five involved critically ill infants who required immediate medical management. Of the 862 included visits, 529 (61 %) were infants’ initial visits included in the study and 333 (39 %) were revisits. Characteristics of the visits and revisits differed between the two study sites with respect to age distribution, reason for visit, and parent/guardian perception of illness severity (Table 1).

**Fig. 1:**
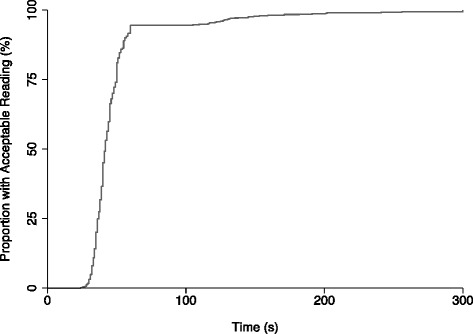
Cumulative proportion of participants with acceptable oxygen saturation (Sp0_2_) values over time, among young infants undergoing routine pulse oximetry at primary health centers in Karachi, Pakistan. Time shown represents pulse oximetry “performance time” (see text for definition)

Acceptable PO readings were obtained in ≤1 and ≤5 min at 94.4 % and 99.8 % of visits, respectively (n = 862). The median PT was 42 s (IQR 37; 50) and median LT was 34 s (IQR 29;40). The proportion of visits with acceptable readings increased rapidly within the first minute; in comparison, the rate of increase in the proportion of infants with acceptable readings during second attempts was relatively slower (Fig. 1).

Among the 48 (of 862) visits at which the first attempt was unsuccessful (i.e., reading was not obtained within 1 min), all (100 %) showed poor quality signals according to the device indicator, 87 % had SpO_2_ values that were fluctuating between at least 2 values, and 40 % did not display a heart rate value.

The distribution of PT differed significantly across sites, even after stratification by infant characteristics (Table 2). In stratified analyses by site, we did not identify any infant characteristics that were significantly associated with PT (Table 2). However, the proportion of acceptable readings obtained in 1 min was significantly greater in the younger age group (0 – 29 days) than in the older age group (30 – 59 days) at Site 2. There was some evidence that acceptable readings were obtained slightly more rapidly as the study progressed, but this did not lead to an increase in the proportion of readings obtained in 1 min (Table 2).

**Table 2:** Pulse oximetry performance time and proportion of acceptable readings obtained within 1 min by site and by infant characteristics among young infants undergoing routine pulse oximetry at primary care clinics in Karachi, Pakistan

	Performance time (seconds), median (IQR)			Acceptable Sp0_2_ value obtained in ≤ 1 min, % (*n*)		
	Site 1	Site 2	*p*	Site 1	Site 2	*p*
Number of visits	481	381	-	481	381	-
Overall	44(39;50)	40(35;46)	<0.0001	94.4(454)	94.5(360)	0.9485
Age (days)^a^						
0 – 29	43(39;50)	40(34;45)	<0.0001	95.5(317)	98.1(209)	0.1011
30 – 59	45(40;51)	39.5(35;49.5)	<0.0001	91.5(137)	89.9(151)	0.5244
*p*	0.0771	0.2127		0.1193	0.0005	
Sex						
Male	44(39;50)	38(35;46)	<0.0001	95.3(244)	95.4(188)	0.9524
Female	44(40;50)	40(35;45)	<0.0001	93.3(210)	93.5(172)	0.9532
*p*	0.7880	0.6813		0.3467	0.4038	
Weight (g)^b^						
980 - 3319	43(39;50)	40(35;45)	<0.0001	94.2(242)	96.5(166)	0.2693
3320 – 5900	45(39.5;50)	39(35;49)	<0.0001	94.6(212)	92.8(194)	0.4339
*p*	0.5961	0.5959		0.8197	0.1164	
Reason for visit^c^						
Well baby	43(39;49)	40(35;44)	0.0225	95.0(94)	95.8(23)	0.8569
Visit foriIllness	44(39;50)	39(35;46)	<0.0001	94.2(360)	94.4(336)	0.9341
*p*	0.2563	0.8513		0.7849	0.7633	
At least one IMCI criterion^d^						
No	44(39;50)	39(34;45)	<0.0001	94.0(331)	95.2(260)	0.5104
Yes	45(39;50)	40(35;47.5)	0.0022	95.4(123)	92.6(100)	0.3701
*p*	0.8151	0.1584		0.5789	0.3078	
Time since start of study period^e^						
First period	45(40;50)	40(36;45)	p < 0.0001	97.1(233)	94.6(176)	0.1983
Second period	43(39;51)	38(33;46)	p < 0.0001	91.7(221)	94.4(184)	0.2830
*p*	0.4007	0.0190		0.0103	0.9099	

^a^Age in days categorized based on median. Correlation between performance time and age was also analyzed as a continuous variable: Site 1- Spearman’s Rho = 0.0927, p = 0.0422; Site 2- Spearman’s Rho = 0.0556, p = 0.2791

^b^Weight in grams categorized based on median. Correlation between performance time and weight was also analyzed as a continuous variable: Site 1- Spearman’s Rho = 0.0625, p = 0.1710; Site 2- Spearman’s Rho = 0.0297, p = 0.5627

^c^ *n* = 861. One reason for visit was listed as “other”, and was excluded from these analyses

^d^Integrated management of childhood illness (IMCI) criteria assessed by the study worker: Temperature < 35.5 °C, temperature > 37.5 °C, respiratory rate ≥ 60, severe lower chest wall in-drawing, no movement or movement only on stimulation, observed convulsions, history of convulsions, history of poor feeding

^e^Date of visit was used to categorize visits into study periods. First period included the first half of measurements for each site, while second period included the second half of measurements for each site. Correlation between performance time and time since start of the study was also analyzed as a continuous variable: Site 1- Spearman’s Rho = −0.0396, p = 0.3865; Site 2- Spearman’s Rho = −0.1809, p = 0.0004

PO was widely accepted by parents/guardians, even at repeated encounters (Table 3). A very small number of parents/guardians perceived that the PO sensor caused pain or discomfort to the infant or that the duration of testing was too long (Table 3). No adverse events were reported.

**Table 3:** Acceptability of pulse oximetry to parents/guardians of young infants undergoing routine pulse oximetry at primary care clinics in Karachi, Pakistan

Concern	Initial Visit	Revisit	*p* ^1^
Were you satisfied with the oxygen test?	*n = 528*	*n = 333*	
Yes	527 (99.8 %)	333 (100 %)	*p* = 1.0
No	1 (0.2 %)	0 (0 %)	
Would you permit the oxygen test to be performed on your baby again in the future?	*n = 512*	*n = 328*	
Yes	509 (99.4 %)	328 (100 %)	*p* = 0.29
No	3 (0.6 %)	0 (0 %)	
Do you believe the oxygen test was useful for the nurses/doctors to check your baby?	*n = 526*	*n = 333*	
Yes	526 (100 %)	333 (100 %)	*p* = 1.0
No	0 (0 %)	0 (0 %)	
Did the parent/guardian express unprompted concerns about:	*n = 527*	*n = 333*	
Pain/discomfort			
Yes	27 (5.1 %)	4 (1.2 %)	*p* = 0.002
No	500 (94.9 %)	329 (98.8 %)	
Heat/burning			
Yes	1 (0.2 %)	0 (0 %)	*p* = 1.0
No	526 (99.8 %)	333 (100 %)	
Sensor was wrapped too tightly			
Yes	0 (0 %)	0 (0 %)	*p* = 1.0
No	527 (100 %)	333 (100 %)	
Test was taking too long			
Yes	0 (0 %)	0 (0 %)	*p* = 1.0
No	527 (100 %)	333 (100 %)	

^**1**^P value for Fisher’s exact test of difference in proportions between initial visits and revisits

**Fig. 2:**
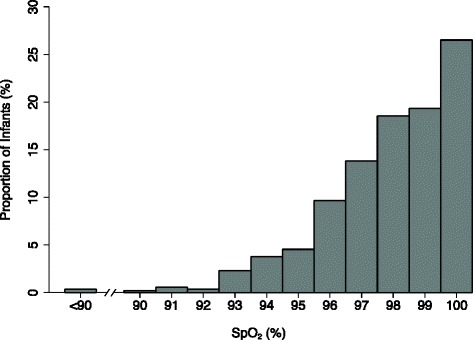
Proportion of infants with measured Sp0_2_ at initial study visits. One infant without an acceptable Sp0_2_ measurement at the initial study visit was excluded from this analysis (*n* = 528)

At 529 initial visits, 528 infants had acceptable SpO_2_ measurements that ranged from 72 % to 100 %, with the majority between 90 and 100 % (median 99 %, IQR 97 % to 100 %) (Fig. 2). There were only 2 initial visits at which SpO_2_ values were <90 %, but 40 (7.6 %) at which SpO_2_ < 95 % (a cut-off commonly used for congenital heart disease screening).

Ten infants with at least one acceptable Sp02 < 92 % at any visit (initial or revisit) contributed a total of 37 visits; SpO2 < 92 % occurred at 13 of 37 (35 %) visits (Table 4). Sp02 was ≥92 % at 2 of the 4 visits at which the first Sp02 < 92 % and there was a repeat attempt at the same visit (Table 4). For example, infant #5 was enrolled in the study at age 3 days, at which time Sp02 was 84 %; however, a repeat measurement during the same visit indicated a Sp0_2_ of 94 %. Further SpO2 measurements at two subsequent revisits were >92 % and the infant was alive and well at the end of follow-up at 2 months of age. Of the 3 (of 10) infants who were not considered to be significantly unwell according to IMCI criteria or physician assessment at the time of first detection of Sp02 < 92 % (Table 4), outcomes were as follows: infant #4, died of unknown cause at 2 weeks of age (described below), infant #7 was reportedly well at 2 months of age, and infant #10 was lost to follow-up (Table 4). Although most of the infants with Sp02 <92 % had good clinical outcomes documented at two months of age, there were two infants with adverse sequelae. Infant #3, who was enrolled in the study during five visits from 10 to 42 days of age, had SpO_2_ <92 % measured repeatedly starting at the third visit (age 37 days). Based on the low Sp02 in combination with the presence of a cardiac murmur, echocardiography was arranged and revealed a diagnosis of tetralogy of Fallot with pulmonary atresia. The infant was referred for specialist pediatric cardiac care and surgical correction. Infant #4 was enrolled at 7 days of age during a well baby visit, at which time SpO_2_ was 84 % and 91 % based on consecutive measurements. The physician assessed the infant as generally well-appearing and not meeting IMCI criteria for referral; however, ten days after participation in the study, the infant died at home of an unknown cause (inconclusive verbal autopsy).

**Table 4:** Oxygen saturation (SpO_2_) and clinical outcomes of participants with at least one SpO_2_ < 92 % at any study visit among young infants undergoing routine pulse oximetry at primary care clinics in Karachi, Pakistan

ID	Age (days)^1^	SpO_2_ attempt #1 (%)^a^	SpO_2_ Attempt #2 (%)^a^	IMCI signs^a,b^	Physician diagnosis^a^	# of study visits^c^	SpO_2_ range (%)^c^	Vital status at 59 days of age
1	3	72	-	RR, NM	Acute respiratory infection	1	N/A	Alive
2	5	73	99	RR, NM	Sepsis	5	91 – 99	Alive
3	37	87	88	RR	Acute respiratory infection, congenital heart disease	5	82 – 93	Alive
4	7	84	91	None	No acute illness	1	N/A	Deceased
5	3	84	94	None	Hyperbilirubinemia	3	97 – 99	Alive
6	1	86	-	RR	Acute respiratory infection	10	93 – 100	Alive
7	0	90	-	None	No acute illness	1	N/A	Alive
8	1	91	-	FVR, RR, LCI, PF	Sepsis	5	94 – 100	Alive
9	14	91	-	RR, LCI	Sepsis	4	97	Alive
10	2	91	-	None	No acute illness	2	97	Lost to follow Up

^a^Refers to first clinic visit at which hypoxemia (SpO_2_ < 92 %) was detected for each infant

^b^Integrated management of childhood illness (IMCI) algorithm signs of very severe disease in a young infant, as detected by the study worker: RR = Respiratory rate ≥ 60; NM = No movement or movement only on stimulation; FVR = Fever; LCI = Severe lower chest wall in-drawing; PF = History of poor feeding

^c^Refers to other clinic visits by the same infant during first 2 months of life

## Discussion

This observational study demonstrated the operational feasibility and parent/guardian acceptability of PO ‘spot checks’ in the routine clinical assessment of young infants at two primary care clinics in low-income communities of Karachi, Pakistan. Using a commercially available handheld device and a sensor-to-infant-first technique, acceptable Sp02 measurements were obtained in nearly all infants in under 1 min, from the time of initiating sensor placement to the display of a stable Sp02 value. The procedure was readily integrated into existing assessment pathways and was well tolerated by the infants. Additionally, parents/guardians had positive views of the technology at nearly all visits. These findings provide a practical basis for further research assessing the clinical effectiveness of PO in the triage and management of young infants in low-income settings.

PO is a painless and portable technology that is widely used in pediatric practice to non-invasively assess and monitor cardiorespiratory function, particularly in perioperative care, emergency departments, critical care units, and in the management of patients with acute or chronic respiratory disease [16]. Despite the availability of PO devices in healthcare facilities, there has not been a strong rationale for its application as a routine ambulatory screening or triage tool because most children and adolescents with hypoxemia will exhibit at least one sign of respiratory distress, cyanosis, or abnormal findings on pulmonary ausculation [17]. However, young infants (<2 months of age) often have subtle and protean presentations of infectious, pulmonary and cardiac diseases [3], and there is particularly poor accuracy of clinical signs for detecting hypoxemia in this age group [4, 18, 19]. Studies in low-income settings have documented Sp02 < 90 % in about one-fifth of hospitalized newborns (irrespective of specific diagnosis), suggesting a higher burden of hypoxemia in this group than among older children with pneumonia, although thresholds for hypoxemia vary by altitude [20] Moreover, studies in Kenya [19] and Papua New Guinea [4] have demonstrated a robust association between hypoxemia and mortality in neonates. Because of the importance of avoiding delays in initiating treatment and appropriate referral of sick young infants, there may be a public health benefit of introducing PO screening in the routine clinical assessment of young infants [5]. In some high-income countries, PO has been recently recommended for the routine screening of all healthy newborns in the early postnatal period (prior to discharge from hospital) for the purpose of detecting critical congenital heart disease [21], However, we are not aware of previous studies of the implementation of routine PO as a screening tool beyond the immediate newborn period.

The widespread implementation of PO in low-income countries remains limited, due in part to practical barriers including lack of access to functioning devices and insufficient training in use of the technology [22]. Where PO devices are available, they are prioritized for use in operating rooms and for rationing supplemental oxygen supplies among patients hospitalized with pneumonia [5]. However, the present study confirmed our hypothesis that modern portable PO technology coupled with a standardized approach by trained personnel would enable this technology to be readily incorporated into the IMCI young infant assessment with minimal burden to the health worker and near universal acceptance by parents/guardians. We did not identify specific infant characteristics (e.g., age) that consistently and substantially affected the primary feasibility metric, PT and success within 1 min. Moreover, there was no correlation between PT at first and second visits by the same infant, indicating the low likelihood that fixed infant characteristics affected PO success. However, significant differences in PT and success by 1 min between the two clinic sites highlighted the importance of considering personnel/system-level factors (e.g., competence of PO operators) that may impact on PO feasibility.

In-hospital implementation of PO has been previously described in The Gambia [23] and Papua New Guinea [11], but we are unaware of any studies of PO feasibility in a primary health care setting in a low-income country. Louis et al. (2014) recently compared two methods of applying PO during resuscitation of newborns in the delivery room at a hospital in northern India, using a Masimo oximeter that uses the same algorithm as the device employed in the present study [13]. With a sensor to infant first approach, they reported high success rates and a median lag time of 16 s. However, a sensor to oximeter first approach, whereby the PO device was left powered-on and attached to the sensor before the sensor was applied to the infant, had a significantly faster lag time [13]. We selected the traditional sensor to infant first approach on the rationale that it prevents the oximeter from averaging ambient noise during the period immediately preceding sensor placement, and would therefore reduce the time to acquire an artifact-free signal [24]. However, Louis et al. argued that modern oximeters have rapid averaging times (~2 s) that avoid such artifacts [13]. Therefore, adoption of a sensor to oximeter approach may have further reduced the performance time in our study setting.

Not unexpectedly, we observed a much lower prevalence of hypoxemia in a primary care setting compared to rates reported in hospital-based studies [20]. Even among the few encounters at which Sp02 < 92 % were detected, the reliability of the majority of those measurements were questioned because of normal repeated measurements or the absence of clinical sequelae. Although we were thus unable to draw firm conclusions about the clinical utility of routine PO from the present study, we did observe a role of PO in the detection of at least one case of major congenital cardiac disease, and potential contributions of PO to the assessment of disease severity in some infants with acute respiratory infections. The clinical effectiveness of routine or targeted PO in this setting would depend on the specific adverse outcomes that the health system in which PO is being undertaken is able to avert. For example, in the absence of a sufficiently developed health system that could accommodate referral of otherwise asymptomatic infants with hypoxemia for echocardiogram (to rule-out congenital heart disease), it may be more rational to target PO to sick infants. Yet, even if PO is reserved for infants with clinically suspected sepsis or respiratory diseases, its value would depend on the efficiency of referral systems and availability of supplemental oxygen supplies.

The protocol for Sp02 spot checks in this study was intended to simulate a practice that could be realistically introduced into a variety of primary care settings. Pulse oximeters were used by first-level personnel (a cadre with less health care training and experience than nurses), but because staff had substantial opportunities for training and practice, and were highly motivated throughout the study, they may not be representative of health care workers who would implement routine pulse oximetry outside of a research context. The small number of study workers who were trained to use pulse oximetry limited the analysis of worker, clinic and system-level factors that may have impacted the performance and feasibility of pulse oximetry in this setting, and which likely accounted for the observed between-site differences. A limitation of the Sp02 interpretation was that the criteria for acceptable readings were based only on information displayed in real-time by the handheld oximeter (e.g., stable Sp02, heart rate, and blip bar signal indicators). Although we used a device that employs a motion-resistant algorithm that has been validated and widely implemented in neonatology [25, 26], it is optimal if operators confirm regular rhythms by monitoring plethysmographic waveforms [16], which would likely not be feasible in routine clinical practice. An inherent threat to the reliability of Sp02 measurement is that PO devices tend to slightly overestimate Sp02 relative to arterial oxygen saturation measured by co-oximetry, and precision and accuracy vary through the Sp02 range [27]. Indeed, the most common SpO2 observed in this study was 100 %, greater than the median 97 % commonly observed at sea level in well neonates [28]. Therefore, it is likely that some infants with mild hypoxemia would not be correctly identified as such using PO. Furthermore, the criteria used in this study to define an acceptable Sp02 (including a stable reading with variation within ±1 % over 10 s) were intended to standardize the procedure in a manner that could be feasibly adopted in routine practice; however, the criteria may have been too restrictive (e.g., excluded readings that were clinically meaningful) and likely included some readings that were unreliable (i.e., met criteria despite an inconsistent underlying waveform). Validation of device-specific ‘acceptability criteria’ for field applications should be a priority of future research.

Future research efforts should aim to establish the conditions under which the introduction of PO into primary care settings improves health outcomes, ideally in conjunction with supplemental oxygen availability. For example, PO may improve clinical outcomes of ill neonates by improving the rational allocation of supplemental oxygen, prompting appropriate referrals to hospital, and identifying infants who warrant further investigations (e.g., echocardiography). Studies should ideally measure PO feasibility and identify context-specific barriers to implementation and scale-up, including long-term robustness of PO devices and components, data that we did not report because of the relatively short duration of this study. In addition to the initial cost of the devices, the high cost of repair and replacement of minor components, such as sensors and cables, is likely to be an important barrier to PO implementation in developing countries [5].

## Conclusions

There is a growing literature suggesting that PO is feasible and improves clinical care of children in low resource settings [5]. The present study adds to this evidence base, by demonstrating the feasibility and acceptability of routine PO in the assessment of young infants at primary care clinics. However, its clinical utility and cost-effectiveness in this context remain to be established.
